# Admission Lysophosphatidic Acid Is Related to Impaired Kidney Function in Acute Aortic Dissection: 2-Year Retrospective Follow-Up Study

**DOI:** 10.3389/fcvm.2022.905406

**Published:** 2022-06-16

**Authors:** Xiaogao Pan, Guifang Yang, Ning Ding, Wen Peng, Tuo Guo, Mengping Zeng, Xiangping Chai

**Affiliations:** ^1^Department of Emergency Medicine, Second Xiangya Hospital, Central South University, Changsha, China; ^2^Emergency Medicine and Difficult Diseases Institute, Second Xiangya Hospital, Central South University, Changsha, China; ^3^Department of Emergency, Changsha Central Hospital, University of South China, Changsha, China

**Keywords:** lysophosphatidic acid, aortic dissection, acute kidney injury, AKI, chronic kidney disease, CKD

## Abstract

**Background:**

Delayed treatment of acute aortic dissection (AAD)-related acute kidney injury (AKI) significantly increases the burden of chronic kidney disease (CKD) and mortality. Lysophosphatidic acid (LPA) is a shared mediator of kidney disease and AAD. Here, we evaluated the relationship between LPA and kidney injury in AAD patients.

**Methods:**

We measured the plasma concentration of LPA in a cohort of 80 patients with AAD. Least Absolute Shrinkage and Selection Operator (LASSO) regression and Logistic regression were used to evaluate the effect and interaction of LPA on AKI. Additive generalized model and penalized spline method were used to describe the non-linear association. Multivariable analyses with the Cox proportional-hazards model were used for subgroup analysis and interaction in LPA and subsequent CKD.

**Results:**

The participant’s average age was 54.27 ± 11.00 years, 68.75% of them were males, and the incidence of AKI was 43.75%. Patients with AKI had higher levels of LPA on admission, and the more significant the increase, the higher the risk of AKI. There was a non-linear positive correlation between admission LPA and AKI, and the premeditated inflection point was 346.33 (μg/dL) through two-piecewise linear regression and recursive algorithm. Subgroup analysis identified a stronger association between admission LPA and AKI in the elder, female and medically treated patients. The incidence of CKD was 22.67% in the 2-year follow-up. Patients with subsequent CKD had higher LPA levels on admission in the follow-up cohort, and a similar interaction trend was also observed through Cox proportional—hazards model.

**Conclusion:**

Admission LPA levels show a non-linear positive correlation with AKI and increase the risk of subsequent CKD, which is more pronounced in elderly, female, and medically treated patients.

## Introduction

Acute aortic dissection (AAD), a life-threatening cardiovascular disease that demands prompt examination and treatment, causes about 1–2% of patients’ deaths per hour in the first 24–48 h (1, 2). Unfortunately, despite improvements in endovascular therapy, open repair, and extracorporeal circulation, there are still 20–30% of patients with shortened survival due to late complications (3, 4). In previous reports, the prevalence of AKI and CKD was noted at 25–55 and 8.5–20% in acute aortic dissection, respectively, which complicates the course of 50–60% patients in intensive care unit and carries significant mortality (5, 6). Consequently, the development of effective approaches to the early recognition of AKI is necessary for reducing the burden of CKD and mortality (7, 8).

Lysophosphatidic acid (LPA) is a bioactive phospholipid, which can exert diverse biological effects in various diseased states of the kidney by activating at least 6 cognate G-protein coupled receptors and its complex network of heterotrimeric G-proteins (9). LPA, mainly produced by activated platelets, is an early biomarker of thrombosis and coagulation initiation in AAD, reflecting the extent of tearing and ischemia (10). Meanwhile, LPA can also increase fibroblast growth factor-23 (FGF23) in acute and chronic kidney injury levels to promote chronic fibrosis, which is closely associated with subsequent CKD and end-stage renal disease (11). Although, an increasing number of studies have been done to explore the effects of LPA metabolism on renal function (9), the association between LPA and AAD-related AKI has not been investigated yet. The aim of this study is to evaluate the association and interaction of LPA with gender, age, Stanford type, and management on AAD-related AKI and subsequent CKD.

## Materials and Methods

### Research Sample and Design

This was a sub-study of a previously conducted retrospective cohort study consisting of 181 patients with suspected AAD treated at the emergency department of the Second Xiangya Hospital of Central South University in Changsha, China from May 2020 and January 2021 (10). We non-selectively and consecutively collected data for all participants and excluded patients who had unruptured aortic intramural hematoma, acute myocardial infarction, and pulmonary embolism. Patients who died within 24 h in perioperative and previous CKD were also excluded due to irregular hemodialysis and indeterminate serum creatinine (sCr). The remaining 80 patients were analyzed for AAD-related AKI and 2-year follow-up. Five of these patients had missing data (lost or death) during follow-up, and the remaining 75 patients were analyzed for long-term CKD. In addition, not included in this study: age < 18 years, tumors, coagulation disorders, and other conditions affecting renal function and serum creatinine (Supplementary Figure 1).

About 3–5 ml of whole blood was taken from the brachial vein and placed in a sodium citrate anticoagulant tube immediately after hospital admission. The samples were centrifuged at 1,000 r/min for 15 min, processed into plasma, and stored at −80°C. The human lysophosphatidic acid kit (CSB-EQ028005HU, Huamei Biological Engineering Co., Ltd., Wuhan, China) was used to measure plasma LPA (10).

### Definitions

AAD was diagnosed based on 2014 ESC guidelines. characterized by symptoms onset-time within 14 days was defined as AAD, dissections involving the ascending aorta are classified as Stanford Type A and those without ascending aorta involvement as Type B, which was confirmed by imaging like computed tomography (CT) or Magnetic resonance imaging (MRI) (12). AKI was defined according to the newest consensus-based KDIGO criteria as follows: small changes in serum creatinine (sCr) (≥ 0.3 μg/dL or 26.5 mmol/l) when they occurred within 48 h or a maximal change in sCr ≥ 1.5 times the baseline value until postoperative day 7 compared with preoperative baseline values or urine volume < 0.5 ml/kg/h for 6 h. Baseline sCr was defined as the value recorded upon emergency room arrival or on the day of admission, and urine output did not be taken into consideration in this study due to its inaccuracy (13). CKD incidence was defined as a 25% reduction in estimated glomerular filtration rate (eGFR) and a fall below 60 ml/min per 1.73 m2, which was determined by the Modification of Diet in Renal Disease (MDRD) equation obtained from creatinine, age, gender, and weight of patients (14, 15). In-hospital mortality was described as all-cause death during admission.

### Variables

Admission LPA for all AAD participants was measured at baseline. Covariates involved demographic (age, gender, smoking, drinking), onset (time, symptoms), comorbidities [hypertension, diabetes, chronic heart disease (CHD), valvular disease, chronic obstructive pulmonary disease (COPD), stroke, obstructive sleep apnea (OSA), and Marfan syndrome], medication history (aspirin, clopidogrel, statins, and hormones), Stanford type (A and B), blood routine [white blood cell (WBC), neutrophil to lymphocyte ratio (NLR), platelet to lymphocyte ratio (PLR), hemoglobin (HGB), and red blood cell distribution width (RDW)], sCr (baseline, maximum, increment), medical therapy (beta-blocker, vasodilator, and analgesic), management (medical, endovascular, and surgical), and outcome (in-hospital death, and CKD).

### Missing Data Addressing

We performed multiple multivariable imputations to address missing data to maximize statistical power and minimize bias. The data analysis had no covariates with missing data. In addition, five imputed datasets with chained equations were created using R-package mice (16). Our multiple imputation of the dataset was mainly based on the following principles: (1) there were no missing data for the primary outcome; (2) replacement of categorical variables was not advisable, as the plausibility is still debated; (3) 5% missingness is suggested as a maximum upper threshold below which multiple imputation provides benefit. Sensitivity analysis found no significant differences between the generated complete data and raw data. Thus, all multivariable analyses results based on the imputed datasets were combined with Rubin’s rules (17, 18).

### Follow-Up and Endpoints

Follow-up was conducted by telephone contact with specialized clinicians. Serum creatinine was tested at the local municipal hospital per 3–6 months after patient discharge. We created an end-point of CKD as we followed for 2 years after patients left the hospital and excluded unexplained deaths. The time to confirm CKD was recorded monthly based on the most recent eGFR results.

### Statistical Analysis

Continuous variables were expressed as mean ± standard deviation. Categorical variables are shown as percentages. Kruskal-Wallis test (skewed distribution), chi-squared test (categorical variables), or ANOVA (one way) were used to analyze normally distributed data, *t*-test and Mann-Whitney *U*-test were used as a *post hoc* analysis. To investigate whether admission LPA correlated with AKI in certain members, statistical analyses were done in three key steps. Step 1: Least Absolute Shrinkage and Selection Operator (LASSO) regression was applied to minimize the potential collinearity of variables measured from the same patient and over-fitting of variables, which was performed to identify the factors of AAD-related AKI (19). Then, three covariates adjustment models were constructed using Logistic regression to show the associated risks. Step 2: non-linearity in admission LPA and AKI was addressed. Fitting of an additive generalized model and penalized spline method (smooth curve) was done. In case any detection of non-linearity was observed, the point of inflection was calculated using a recursive algorithm, and a linear two-piece regression was constructed. This was done on the inflection point for both sides. For the likelihood log-ratio test, the best fit model was checked on the *P*-values. Step 3: a stratified linear regression model was used for subgroup analyses. Continuous variables were changed to categorical variables as stated in the clinical tertile (cut point) and an interaction test was done. A sensitivity study was used to confirm the stoutness of data analysis that converted admission LPA to a categorical variable and the trend’s *p*-value calculated. The aim was to detect the likelihood of non-linearity and to affirm LPA admission results as a continuous variable. Multivariable analyses with the Cox proportional-hazards model were used for subgroup analysis and interaction in admission LPA and CKD. EmpowerStats^1^ (X and Y Inc. Solutions, Boston, MA) and R version 4.0.5^2^ were used for statistical analyses. *P* ≤ 0.05 was considered statistically significant.

## Results

### Characteristics of Baseline

**FIGURE 1:**
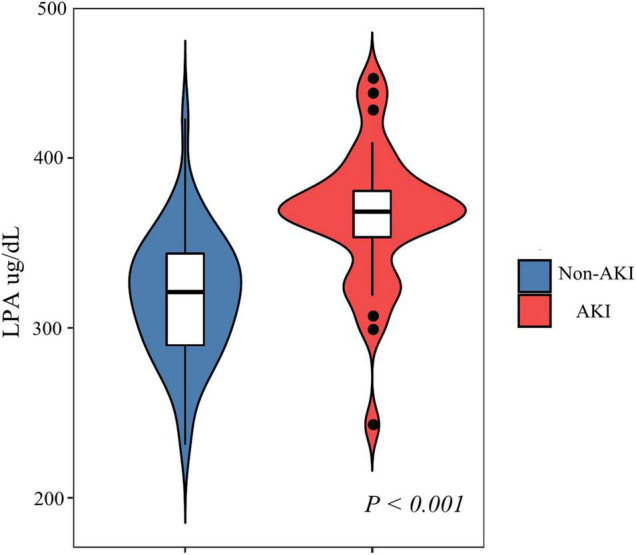
Comparison of admission LPA stratified by AKI.

Based on exclusion and inclusion criteria, 80 participants were included. The baseline characteristics are shown in Table 1 based on the tertiles of LPA. The participant’s average age was 54.27 ± 11.00 years and 68.75% of them were males. No statistically significant differences were observed for age, gender, symptoms, onset time, comorbidities, medication history, baseline lab test, and baseline serum creatinine (*P* > 0.05). Medical therapy to control pain, heart rate, and blood pressure was also not statistically different (*P* > 0.05). Contributors with the uppermost group of admission LPA (Q3) had higher values in hypertension, Stanford type A, maximum and incremental sCr, and incidence of AKI and CKD. Similar patterns were observed for in-hospital mortality in Q2 groups and Stanford type B in Q1groups. Supplementary Table 1 shown that the incidence of AKI was 43.75% in this study, and the AKI group had a higher probability of hypertension and diabetes compared to the non-AKI group, which was more likely to have high levels of admission LPA (Figure 1).

**TABLE 1:** Baseline characteristics of the patients about the tertile admission LPA.

Variables	Q1 (231.12–321.09)	Q2 (321.63–362.01)	Q3 (362.22–446.79)	*P*-value
Number	27	26	27	
Age, year	54.44 ± 10.35	52.62 ± 12.71	55.70 ± 10.01	0.596
**Gender**				0.424
Male	20 (74.07%)	19 (73.08%)	16 (59.26%)	
Female	7 (25.93%)	7 (26.92%)	11 (40.74%)	
**Symptoms**				0.252
Chest pain	16 (59.26%)	17 (65.38%)	22 (81.48%)	0.191
Bellyache	9 (33.33%)	6 (23.08%)	2 (7.41%)	0.064
Syncope	0 (0.00%)	2 (7.69%)	1 (3.70%)	0.316
Other	2 (7.41%)	1 (3.85%)	2 (7.41%)	0.827
Onset time, h	10.56 ± 5.45	10.19 ± 8.60	11.41 ± 5.71	0.795
**Comorbidities**				
Hypertension	11 (40.74%)	14 (53.85%)	20 (74.07%)	0.045
Diabetes	2 (7.41%)	3 (11.54%)	6 (22.22%)	0.265
CHD	4 (14.81%)	2 (7.69%)	4 (14.81%)	0.666
Valvular disease	1 (3.70%)	1 (3.85%)	3 (11.11%)	0.440
COPD	2 (7.41%)	2 (7.69%)	2 (7.41%)	0.986
Stroke	2 (7.41%)	1 (3.85%)	3 (11.11%)	0.604
OSA	4 (14.81%)	5 (19.23%)	5 (18.52%)	0.901
Marfan syndrome	1 (3.70%)	1 (3.85%)	0 (0.00%)	0.769
Smoking	14 (51.85%)	14 (53.85%)	10 (37.04%)	0.404
Drinking	7 (25.93%)	4 (15.38%)	5 (18.52%)	0.614
**Medication history**				
Aspirin	7 (25.93%)	5 (19.23%)	4 (14.81%)	0.590
Clopidogrel	3 (11.11%)	1 (3.85%)	2 (7.41%)	0.604
Statins	3 (11.11%)	4 (15.38%)	4 (14.81%)	0.886
Hormones	1 (3.70%)	2 (7.69%)	1 (3.70%)	0.745
**Stanford type**				0.005
A	11 (40.74%)	20 (76.92%)	21 (77.78%)	
B	16 (59.26%)	6 (23.08%)	6 (22.22%)	
**Baseline lab test**				
WBC, 10^9^/L	11.56 ± 2.87	12.28 ± 4.29	12.81 ± 4.66	0.522
HGB, g/L	127.22 ± 15.04	124.08 ± 14.99	127.96 ± 14.64	0.605
RDW, %	13.54 ± 1.49	13.62 ± 1.45	13.38 ± 1.13	0.800
NLR	12.08 ± 6.22	13.81 ± 7.82	14.12 ± 8.51	0.570
PLR	173.89 ± 68.58	184.31 ± 95.84	194.40 ± 91.79	0.683
LPA, μg/dL	291.59 ± 24.00	340.78 ± 12.53	388.11 ± 31.09	< 0.001
**sCr,** μ**mol/l**				
Baseline	71.43 ± 20.14	75.57 ± 33.57	82.35 ± 33.92	0.403
Maximum	80.37 ± 34.86	98.65 ± 48.66	149.48 ± 69.67	< 0.001
Increment	8.94 ± 27.36	23.04 ± 31.29	67.10 ± 46.53	< 0.001
**Medical therapy**				
Beta-blocker	25 (92.59%)	26 (100%)	26 (96.30%)	0.760
Vasodilator	27 (100%)	25 (96.15%)	25 (92.59%)	0.763
Analgesic	25 (92.59%)	24 (92.31%)	25 (92.59%)	0.998
**Management**				< 0.001
Medical	1 (3.70%)	2 (7.69%)	4 (14.81%)	0.343
Endovascular	15 (55.56%)	6 (23.08%)	3 (11.11%)	0.001
Surgical	11 (40.74%)	18 (69.23%)	20 (74.07%)	0.025
**Outcome**				
AKI	4 (14.81%)	8 (30.77%)	23 (85.19%)	<0.001
In-hospital death	2 (7.41%)	9 (34.62%)	9 (33.33%)	0.034
CKD^#^	0 (0.00%)	3 (11.54%)	14 (51.85%)	<0.001

*Data are presented as n (%) or mean ± standard deviation. CHD, chronic heart disease; COPD, chronic obstructive pulmonary disease; OSA, obstructive sleep apnea; WBC, white blood cell; HGB, hemoglobin; NLR, neutrocyte lymphocyte ratio; RDW, red cell volume distribution width; PLR, platelet lymphocyte ratio; LPA, lysophosphatidic acid; sCr, serum creatinine; AKI, acute kidney injury; CKD, chronic kidney disease. # represents 75 people participating in the follow-up.*

### Adjusted and Unadjusted Models

All variables measured at hospital (Table 1) were included in the LASSO regression. After LASSO regression selection (Supplementary Figure 2), 10 variables remained significant predictors of AKI, including clinical features and test results: age, hypertension, diabetes, Marfan syndrome, COPD, medication history of aspirin, Stanford type, WBC, LPA, and Management.

We defined the above 10 variables other than LPA as covariates affecting the risk of AKI in AAD patients. We constructed three models to analyze the independent effects of LPA on AKI (univariate and multivariate) based on Logistic regression model. The odds ratio (OR) and 95% confidence intervals were listed in Table 2. In the full model (model II), after adjusting for all covariates, for every 1, 10, 20, 30 μg/dL, and one standard deviation rise in admission LPA, risk of AKI increased, respectively, by 5% (1.05, 95% CI 1.02, 1.08), 67% (1.67, 95% CI 1.27, 2.21), 180% (2.80, 95% CI 1.60, 4.88), 368% (4.68, 95% CI 2.03, 10.79), and 986% (10.86, 95% CI 2.98, 39.52). We also converted admission LPA from a continuous variable to a categorical variable (Tertiles). P for the trend of admission LPA with categorical variables in the fully adjusted model was constant with the result with admission LPA as a constant variable. However, when the admission LPA enters the fully adjusted model as a categorical variable, the trend of the effective value in the different admission LPA group is non-equidistant. Based on these non-equidistant changes in effect size, there may be a non-linear relationship between admission LPA and AKI.

**TABLE 2:** Relationship between LPA and risk of AKI in different models.

Exposure	Crude model (OR, 95%CI, P)	Model I (OR, 95%CI, P)	Model II (OR, 95%CI, P)
LPA (μg/dL, each 1 increment)	1.03 (1.02, 1.05) <0.001	1.03 (1.02, 1.05) <0.001	1.05 (1.02, 1.08) <0.001
LPA (μg/dL, each 10 increment)	1.40 (1.19, 1.66) <0.001	1.41 (1.18, 1.67) <0.001	1.67 (1.27, 2.21) <0.001
LPA (μg/dL, each 20 increment)	1.97 (1.41, 2.75) <0.001	1.98 (1.40, 2.79) <0.001	2.80 (1.60, 4.88) <0.001
LPA (μg/dL, each 30 increment)	2.77 (1.68, 4.57) <0.001	2.78 (1.66, 4.67) <0.001	4.68 (2.03, 10.79) <0.001
LPA (μg/dL, each SD increment)	4.83 (2.23, 10.47) <0.001	4.86 (2.18, 10.82) <0.001	10.86 (2.98, 39.52) <0.001
**LPA (μg/dL) (tertile)**			
T1	Ref	Ref	Ref
T2	2.56 (0.66, 9.85) 0.173	2.79 (0.70, 11.05) 0.144	4.64 (0.76, 18.30) 0.096
T3	13.06 (7.37, 28.42) <0.001	13.78 (7.72, 30.19) <0.001	15.98 (6.17, 27.91) 0.003
P for trend	0.011	0.013	0.018

***Crude Model** adjusted for none. **Model I** adjusted for age and gender. **Model II** adjusted for age, gender, hypertension, diabetes, Marfan syndrome, COPD, medication history of aspirin, Stanford type, WBC, LPA, and management. COPD, chronic obstructive pulmonary disease; WBC, white blood cell; LPA, lysophosphatidic acid.*

### Non-linear Relationships Between Lysophosphatidic Acid and Acute Kidney Injury

**FIGURE 2:**
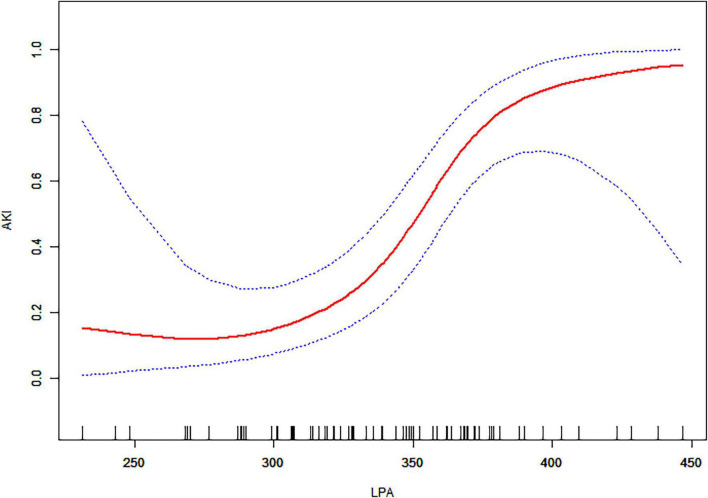
The relationship between admission LPA and AKI. A non-linear association between admission LPA levels and AKI was found in a generalized additive model (GAM). The solid red line represents the smooth curve fit between variables. Blue bands represent the 95% confidence interval from the fit. All adjusted for age, gender, hypertension, diabetes, Marfan syndrome, COPD, medication history of aspirin, Stanford type, WBC, and management.

We examined the non-linear correlation between admission LPA and AKI (Figure 2 and Table 3). The smooth curve results revealed the non-linearly positively correlated after adjusting for the above 9 covariates. We fit the association between admission LPA and AKI using linear regression model and two-piecewise linear regression model, respectively. The *p*-value for the log-likelihood ratio test was 0.014, which indicated dual piecewise linear regression was more appropriate for fitting the association between admission LPA and AKI since it perfectly represents the association. The premeditated inflection point was 346.33 (μ/dL) through two-piecewise linear regression and recursive algorithm. On the left side of the inflection point (LPA ≤ 346.33 μg/dL), the effect size and 95% CI was 1.02 and 0.98–1.05, without statistically significant (*P* > 0.05). On the right side of the inflection point (LPA > 346.33 μg/dL), the effect size and 95% CI was 1.15 (1.05–1.26), which was statistically significant (*P* = 0.003).

**TABLE 3:** The results of the two-piecewise linear model.

	AKI (OR, 95%CI)	*P-*value
Fitting model by standard linear regression	1.05 (1.02, 1.08)	<0.001
**Fitting model by two-piecewise linear regression**		
Inflection point of LPA (μg/dL)	346.33	
≤346.33	1.02 (0.98, 1.05)	0.387
>346.33	1.15 (1.05, 1.26)	0.003
Difference in two-piecewise effect	1.13 (1.02, 1.26)	0.024
P for log-likelihood ratio test		0.014

*Adjusted: age, gender, hypertension, diabetes, Marfan syndrome, COPD, medication history of aspirin, Stanford type, WBC, LPA, and management.*

*COPD, chronic obstructive pulmonary disease; WBC, white blood cell; LPA, lysophosphatidic acid.*

### Subgroup Analysis and Interaction Between Lysophosphatidic Acid and Acute Kidney Injury

**FIGURE 3:**
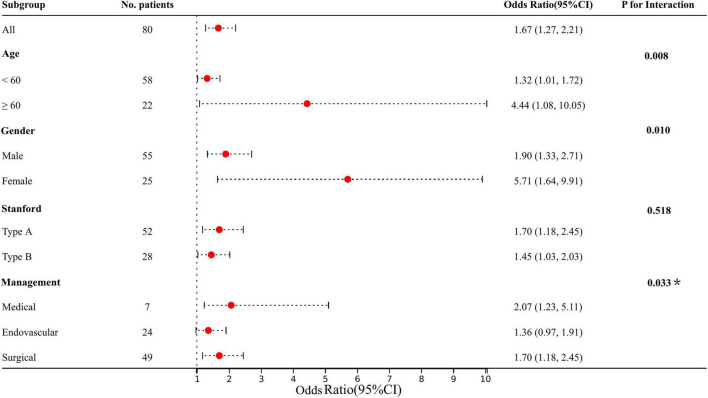
Results of subgroup analysis and interaction analysis between LPA and AKI (LPA per 10 increments). All adjusted for age, gender, hypertension, diabetes, Marfan syndrome, COPD, medication history of aspirin, Stanford type, WBC, and management. *P-value for the management is for the comparison of patients treated medically with patients treated endovascularly.

The predetermined covariates were gender (male vs. female), age (< 60 years vs. ≥ 60 years), Stanford type (A vs. B), and management (medical vs. endovascular vs. open repair) according to clinical guidelines and previous studies. We evaluated interactions between the four factors and LPA (per 10 μ/dL) with a stepwise procedure for multivariate analysis (Figure 3). The results revealed that the deviations in these populations were more pronounced: elder (vs. 1.32 with young), female (vs. 1.90 with male), and medical (vs. 1.36 with endovascular) (all p for interaction < 0.05). We still observed a higher risk of AKI with Stanford type A and surgical, although the interaction with LPA was not significant.

### Two-Year Follow-Up: Lysophosphatidic Acid Increases the Risk of Chronic Kidney Disease

**FIGURE 4:**
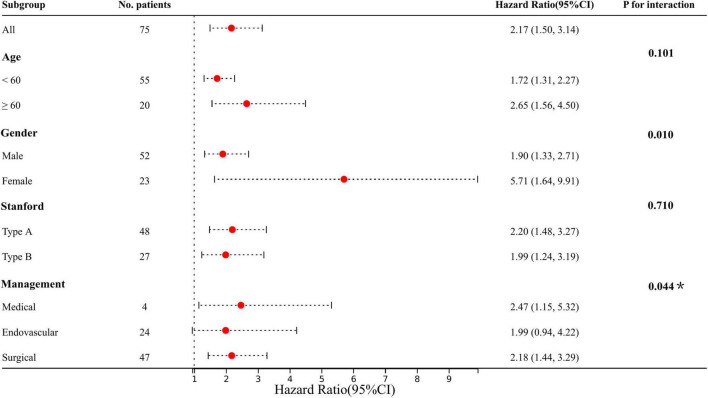
Results of subgroup analysis and interaction analysis between LPA and CKD (LPA per 10 increments). Adjusted: age, gender, hypertension, diabetes, CHD, valvular disease, COPD, stroke, OSA, Marfan syndrome, smoking, drinking, medication history of aspirin, clopidogrel, statins, and hormones. *P-value for the management is for the comparison of patients treated medically with patients treated endovascularly.

To verify the association between admission LPA and the risk of subsequent CKD, a total of 75 participants were analyzed for long-term CKD with a median follow-up of 17.25 months, of whom 17 (22.67%) developed CKD. Patients with AKI were > 3 times more likely to develop CKD than those who do not (17.33 vs. 5.33%). Admission LPA levels in CKD patients were shown in Supplementary Figure 3. Similar to the performance of AKI, after Cox proportional-hazards model adjusting for comorbid factors, subgroup analysis and interaction showed that LPA also increased the risk of CKD, and interacted with age, gender, and management (P for interaction < 0.05) (Figure 4).

## Discussion

Our findings in the present study were summarized as follows: (1) patients with AKI had higher levels of LPA on admission, and the more significant the increase, the higher the risk of AKI; (2) there was a non-linear positive correlation between admission LPA and AKI, and the premeditated inflection point was 346.33 (μg/dL) through two-piecewise linear regression and recursive algorithm; (3) subgroup analysis identified a stronger association between admission LPA and AKI in the elder, female and medically treated patients; (4) patients with subsequent CKD had higher LPA levels on admission in the follow-up cohort, and a similar interaction trend was also observed through Cox proportional-hazards model.

The occurrence of AKI in AAD has important implications, and the adverse effect of AKI in the perioperative period has been well described for patients with AAD previously (20–22). The incidence, trends, risk factors, management, and prognosis of AAD-related AKI have been revealed in a series of studies (6, 20). Regardless of treatment strategy and definition of AKI, studies have shown that AAD-related AKI is associated with hypertension, diabetes, advanced age, gender, and arterial malperfusion, which is consistent with our findings (23–25). But non-linear correlations, subgroup analyses, and interactions have not been well characterized in other studies and are the focus of this investigation. The application of LASSO regression can mainly avoid overfitting and multicollinearity, but its limitation is mainly that it is difficult to obtain an estimate of the standard error, which affects the calculation of effect size and confidence interval, and needs to be noted in future research. The non-linear correlation, between admission LPA and the risk of AAD-related AKI, may suggest that the risk corresponding to different LPA levels may not be a simple “linear superposition,” but needs to take into account the inflection point or threshold effect, which may provide some references to explore the pathophysiology of LPA in patients with AAD-related AKI in the future. We also found higher in-hospital mortality in the Q2 and Q3 groups (Table 1), which may revealed that high levels of LPA may reflect the severity of aortic dissection, extent of intimal tear, and progression of adverse complications, but more verification is required.

The mechanism of elevated LPA in AAD patients has previously been described in our study (10). To summarize in brief, the pathophysiology for elevation is platelet activation, thrombosis, ischemia/reperfusion injury, and inflammatory responses, which also underlie the pathogenesis of AAD-related AKI. Multiple findings highlighted the significant role that arterial malperfusion and ischemic-related inflammatory responses play in the development of AKI (6, 26). Consistently, as important mediators, LPA produced an overall beneficial effect to protect the cells in the kidney if exogenous injection in conditions of AKI (9, 27, 28). For example, Mirzoyan et al. observed that exogenous LPA exerted a protective action against renal inflammation and injuries caused by bacterial endotoxemia through reducing the upregulation of inflammatory cytokines [IL-6, TNFα, monocyte chemoattractant protein-1 (MCP-1)] (29). de Vries et al. also found that LPA prevented renal ischemia-reperfusion injury by inhibition of apoptosis and complement activation through reducing cleaved caspase-7 levels (27). However, it is possibly that the continuous damaging to renal function of ischemia/reperfusion and inflammatory responses outweigh the overall protection of LPA in AAD-related AKI (6, 20). Under the conditions of aortic-tear-related ischemia/reperfusion and inflammatory responses, any compensatory regulatory mechanisms may be weak and inadequate in the AAD-related AKI until surgical repaired. Elevated LPA may reflect severe aortic intima tear, and may also reveal the degree of renal injury in AAD-related AKI. It is certain that LPA itself will not trigger AKI, by reason that the incidence of AKI remains low in acute myocardial infarction and pulmonary embolism with high LPA levels from clinical perspective (10). As expected, our results highlighted the association of plasma LPA levels with the risk of AAD-related AKI, rather than the regulatory mechanisms. Those, with high levels of LPA, may also incarnate more severe ischemic/perfusion and inflammatory responses, e.g., the elderly, female, and medically treated patients in this study, which is consistent with clinical practice. The elderly, with multiple comorbidities such as hypertension, diabetes, and arteriosclerosis, are prone to poor basic renal function. Females are more prone to comorbid connective tissue diseases, and medically treated patients are often susceptible to unstable blood pressure, aggravated aortic tear, and deteriorated renal ischemia.

Our findings revealed that AKI patients are more likely to develop subsequent CKD in AAD. In previous reports, the prevalence of CKD was estimated as being 8.5–20% of patients, with many comorbid factors, such as hypertension, arteriosclerosis, inflammation, etc. (14, 30, 31). Whether in animal experiments or clinical studies, researches have shown that LPA is a potential factor in the pathogenesis of CKD. Although the pathogenesis of CKD is complex, evidences show that exogenous glycerol-3-phosphate (G-3-P) stimulated bone and bone marrow fibroblast growth factor-23 (FGF-23) production through local G-3-P acyltransferase mediated (GPAT-mediated) LPA synthesis (11, 32). FGF-23 levels increasing may contribute directly to the transition of AKI to chronicity, through profibrotic effects and effects on renal immune function damage, and LPA plays an important role throughout the process by activating LPA receptor 1 (11, 25, 33, 34). There is also evidence that blockade of the LPA1/3 receptor activity attenuated glomerular injury by reducing podocyte loss, prevented a decline in glomerular filtration rate (GFR) and reduced proteinuria (35, 36). The research on the metabolic mechanism of LPA in CKD has never stopped, and the findings have also been described in detail in the previous literature (9). Regardless of basic or clinical research, it seems difficult to use LPA to quantify the risk of CKD, despite a strong association. Consequently, our study attempted to quantify this risk to provide some clinical evidence. Although more verification will be needed, we still observed that high LPA levels increase the risk of subsequent CKD and interacted with age, gender, and treatment strategy.

These findings may provide some insight that AAD patients with high LPA levels at admission may be at higher risk for AKI and subsequent CKD. A major drawback of the current study is that urine output data was not collected due to lack of data and difficulties in recording in the electronic medical record system, which needs to be addressed in future studies. Whereas, early subtle changes in renal function and urine output of patients still need to be captured for evaluation and grading. In the management strategy of these patients, clinicians should minimize the ischemic time of visceral organs and the kidneys, avoid natriuretic peptide, dopamine, or mannitol for the treatment, and start renal replacement therapy promptly (6).

## Limitations

The study still has some limitations. First, a large-scale prospective multicenter study is needed to confirm the generalizability of the findings. Second, from the results of smooth curve fitting, although LPA < 250 (μg/dL) showed a weak negative correlation with the risk of AKI, the sample size was too small (only 3 cases), and the statistical significance was not clear. Further validation should be done in future studies with an increased sample size. Third, constrained by our national medical system, follow-up eGFR results may delay the diagnosis of CKD, even if we try to minimize bias. Forth, limited by the electronic medical record system, dissections involving renal arteries were not differentiated, and time factors were also not included such as surgical clamping, endovascular procedures, and duration of treatment, etc. Besides, other diseases (ovarian cancer) can also increase LPA levels (37). Finally, AKI caused by contrast-induced nephropathy cannot currently be excluded as all patients underwent CTA, although the incidence is quite low in patients without renal diseases.

## Conclusion

Admission LPA levels show a non-linear positive correlation with AKI and increase the risk of subsequent CKD, which is more pronounced in elderly, female, and medically treated patients.

## Data Availability Statement

The original contributions presented in this study are included in the article/Supplementary Material, further inquiries can be directed to the corresponding author/s.

## Ethics Statement

The studies involving human participants were reviewed and approved by the Hospital Institutional Review Board of the Second Xiangya Hospital. Written informed consent for participation was not required for this study in accordance with the national legislation and the institutional requirements.

## Author Contributions

XP and XC drafted, revised, and reviewed the article. XP, GY, and ND conducted the statistical analysis and reviewed and revised the manuscripts. WP, TG, and MZ organized the database. All authors significantly contributed to the conception, study design, execution, data acquisition, analysis, and interpretation, approved the final version, and agreed on the journal and were responsible for this study.

## Conflict of Interest

The authors declare that the research was conducted in the absence of any commercial or financial relationships that could be construed as a potential conflict of interest.

## Publisher’s Note

All claims expressed in this article are solely those of the authors and do not necessarily represent those of their affiliated organizations, or those of the publisher, the editors and the reviewers. Any product that may be evaluated in this article, or claim that may be made by its manufacturer, is not guaranteed or endorsed by the publisher.

## Footnotes

1
http://www.empowerstats.com

2
http://www.r-project.org

## Supplementary Material

Supplementary Figure 1 Flow chart of patient enrollment.

Supplementary Figure 2 Feature selection using the least absolute shrinkage and selection operator (LASSO) binary logistic regression model. **(A)** LASSO coefficient profiles of all variables. **(B)** Tuning parameter (λ) selection in the LASSO model used 10-fold cross-validation *via* minimum criteria.

Supplementary Figure 3 Comparison of admission LPA stratified by CKD.
