# Supplementary material for: Admission Lysophosphatidic Acid Is Related to Impaired Kidney Function in Acute Aortic Dissection: 2-Year Retrospective Follow-Up Study
Source: Front Cardiovasc Med. 2022 Jun 16;9:905406. doi: 10.3389/fcvm.2022.905406 (PMC9246270; doi:10.3389/fcvm.2022.905406)
Supplement: Supplementary Figure 1 — Flow chart of patient enrollment. [file Data_Sheet_1.docx]

**Supplementary materials**

**Table S1. Baseline characteristics of the patients stratified by AKI.**

| **Variables** | **All** | **Non-AKI** | **AKI** | **P-value** |
| --- | --- | --- | --- | --- |
| Number | 80 | 45(56.25%) | 35(43.75%) |  |
| Age, year | 54.27 ± 11.00 | 52.78 ± 11.87 | 56.20 ± 9.58 | 0.169 |
| **Gender** |  |  |  | 0.605 |
| Male | 55 (68.75%) | 32 (71.11%) | 23 (65.71%) |  |
| Female | 25 (31.25%) | 13 (28.89%) | 12 (34.29%) |  |
| **Symptoms** |  |  |  | 0.818 |
| Chest pain | 55 (68.75%) | 29 (64.44%) | 26 (74.29%) | 0.346 |
| Bellyache | 17 (21.25%) | 11 (24.44%) | 6 (17.14%) | 0.428 |
| Syncope | 3 (3.75%) | 2 (4.44%) | 1 (2.86%) | 0.711 |
| Other | 5 (6.25%) | 3 (6.67%) | 2 (5.71%) | 0.861 |
| Onset time, h | 10.72 ± 6.65 | 10.76 ± 7.74 | 10.69 ± 5.00 | 0.963 |
| **History of** |  |  |  |  |
| Hypertension | 45 (56.25%) | 19 (42.22%) | 26 (74.29%) | 0.004 |
| Diabetes | 11 (13.75%) | 3 (6.67%) | 8 (22.86%) | 0.037 |
| CHD | 10 (12.50%) | 5 (11.11%) | 5 (14.29%) | 0.670 |
| Valvular disease | 5 (6.25%) | 2 (4.44%) | 3 (8.57%) | 0.449 |
| COPD | 6 (7.50%) | 2 (4.44%) | 4 (11.43%) | 0.239 |
| Stroke | 6 (7.50%) | 2 (4.44%) | 4 (11.43%) | 0.239 |
| OSA | 14 (17.50%) | 8 (17.78%) | 6 (17.14%) | 0.941 |
| Marfan syndrome | 2 (2.50%) | 2 (4.44%) | 0 (0.00%) | 0.207 |
| Smoking | 38 (47.50%) | 23 (51.11%) | 15 (42.86%) | 0.463 |
| Drinking | 16 (20.00%) | 10 (22.22%) | 6 (17.14%) | 0.573 |
| **Medication history** |  |  |  |  |
| Aspirin | 16 (20.00%) | 11 (24.44%) | 5 (14.29%) | 0.260 |
| Clopidogrel | 6 (7.50%) | 3 (6.67%) | 3 (8.57%) | 0.748 |
| Statins | 11 (13.75%) | 5 (11.11%) | 6 (17.14%) | 0.437 |
| Hormones | 4 (5.00%) | 3 (6.67%) | 1 (2.86%) | 0.628 |
| **Stanford type** |  |  |  | 0.906 |
| A | 52 (65.00%) | 29 (64.44%) | 23 (65.71%) |  |
| B | 28 (35.00%) | 16 (35.56%) | 12 (34.29%) |  |
| **Baseline lab test** |  |  |  |  |
| WBC, 10^9^ /L | 12.22 ± 3.99 | 11.76 ± 3.64 | 12.81 ± 4.40 | 0.246 |
| HGB, g/L | 126.45 ± 14.79 | 126.02 ± 15.08 | 127.00 ± 14.62 | 0.771 |
| RDW, % | 13.51 ± 1.35 | 13.57 ± 1.39 | 13.44 ± 1.32 | 0.682 |
| NLR | 13.33 ± 7.53 | 12.75 ± 7.10 | 14.07 ± 8.10 | 0.443 |
| PLR | 184.20 ± 85.44 | 176.01 ± 75.63 | 194.73 ± 96.72 | 0.334 |
| LPA, ug/dl | 340.15 ± 46.37 | 319.16 ± 36.39 | 367.14 ± 44.14 | <0.001 |
| **SCr, umol/l** |  |  |  |  |
| Baseline | 76.46 ± 29.83 | 71.66 ± 19.20 | 82.63 ± 38.99 | 0.103 |
| Maximum | 109.64 ± 60.18 | 73.46 ± 17.52 | 156.14 ± 63.72 | <0.001 |
| Increment | 33.15 ± 43.55 | 2.00 ± 11.37 | 73.20 ± 36.12 | <0.001 |
| **Medical therapy** |  |  |  |  |
| Beta-blocker | 77 (96.25%) | 44 (97.78%) | 33 (94.29%) | 0.212 |
| Vasodilator | 77 (96.25%) | 43 (95.56%) | 34 (97.14%) | 0.603 |
| Analgesic | 74 (92.50%) | 41 (91.11%) | 33 (94.29%) | 0.096 |
| **Management** |  |  |  | 0.746 |
| Medical | 7 (8.75%) | 4 (8.89%) | 3 (8.57%) | 0.960 |
| Endovascular | 24 (30.00%) | 15 (33.33%) | 9 (25.71%) | 0.461 |
| Surgical | 49 (61.25%) | 26 (57.78%) | 23 (65.71%) | 0.470 |
| **Outcome** |  |  |  |  |
| In-hospital death | 20 (25.00%) | 10 (22.22%) | 10 (28.57%) | 0.515 |
| CKD^#^ | 17 (22.67%) | 4 (5.33%) | 13 (17.33%) | 0.002 |

Data are presented as n (%) or mean ± standard deviation. **Abbreviations:** CHD, chronic heart disease; COPD, chronic obstructive pulmonary disease; OSA, obstructive sleep apnea; WBC, white blood cell; HGB, haemoglobin; NLR, neutrocyte lymphocyte ratio; RDW, red cell volume distribution width; PLR, platelet lymphocyte ratio; LPA, lysophosphatidic acid; sCr, serum creatinine; AKI, acute kidney injury; CKD, chronic kidney disease. # represents 75 people participating in the follow-up.

**Table S2. Comparison of plasma LPA (ug/dL) concentrations in AAD patients by age and gender**

|  | No. | Mean | SD | Lower quartile | Median | Upper quartile | P value* |
| --- | --- | --- | --- | --- | --- | --- | --- |
| **Total** | 80 | 339.41 | 44.11 | 307.23 | 340.18 | 368.68 |  |
| **Age** |  |  |  |  |  |  | 0.369 |
| < 60 | 58 | 339.98 | 40.23 | 310.25 | 338.88 | 367.88 |  |
| ≥ 60 | 22 | 349.28 | 43.36 | 313.90 | 359.57 | 378.94 |  |
| **Gender** |  |  |  |  |  |  | 0.043 |
| Male | 55 | 336.30 | 39.47 | 306.61 | 333.17 | 365.94 |  |
| Female | 25 | 356.25 | 41.91 | 362.01 | 338.22 | 378.94 |  |

*Mann-Whitney test for difference.


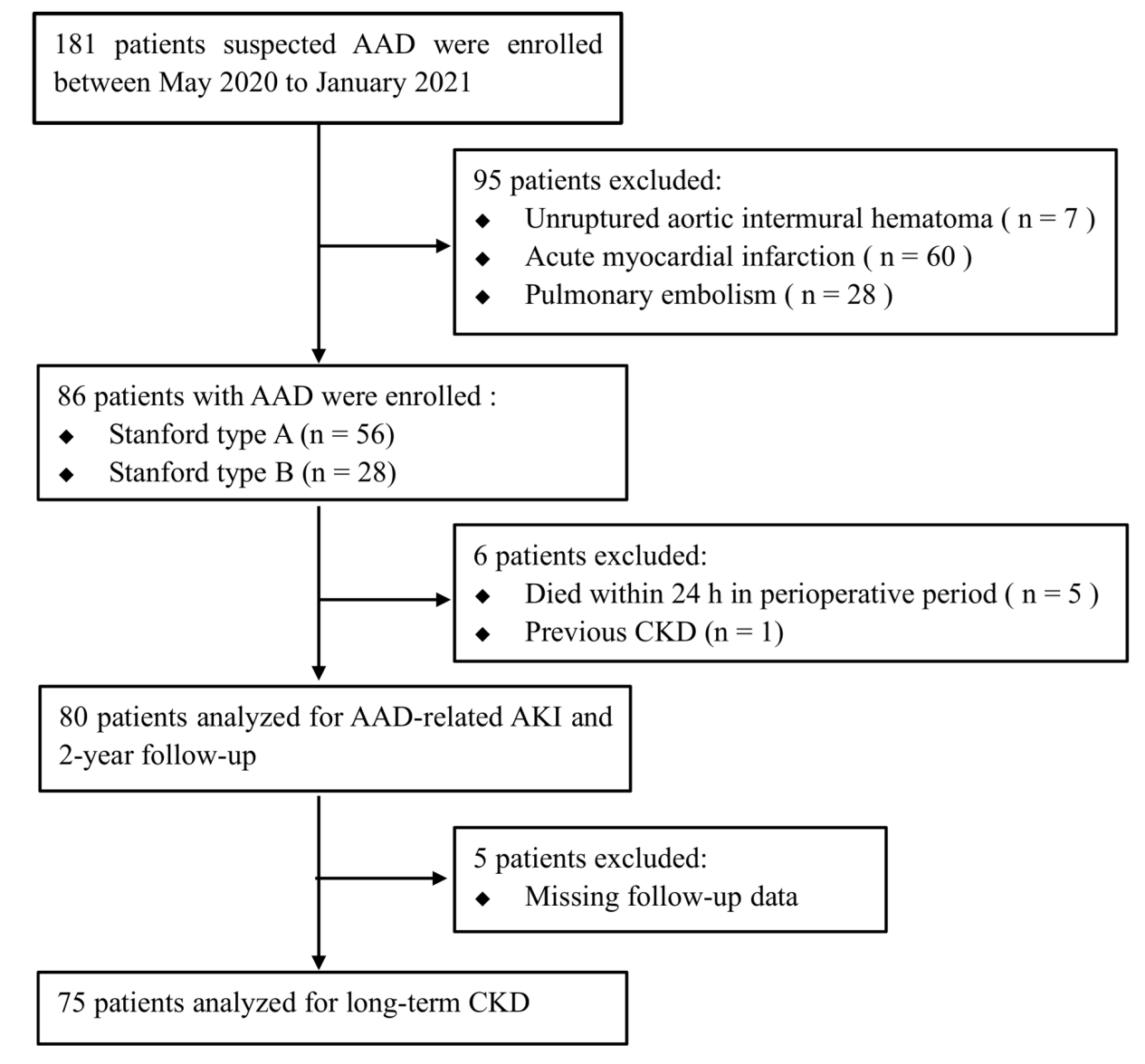


**Figure S1**. Flow chart of patient enrollment.







1. (B)

**Figure S2**. Feature selection using the least absolute shrinkage and selection operator (LASSO) binary logistic regression model. (A) LASSO coefficient profiles of all variables. (B) Tuning parameter (λ) selection in the LASSO model used 10-fold cross-validation via minimum criteria.


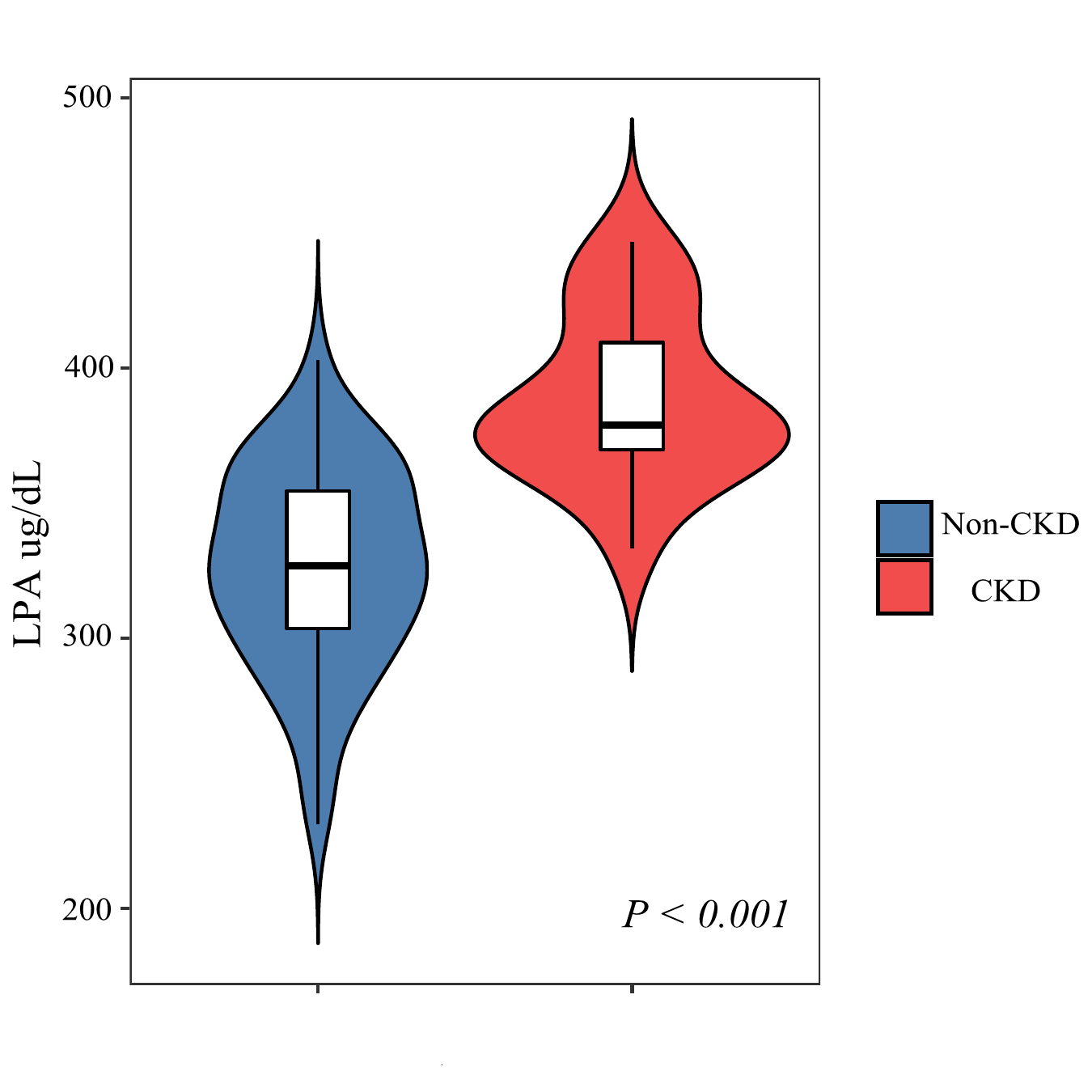


**Figure S3**. Comparison of admission LPA stratified by CKD.
